# Time-trend analysis of the center frequency of the intrinsic mode function from the Hilbert–Huang transform of electroencephalography during general anesthesia: a retrospective observational study

**DOI:** 10.1186/s12871-023-02082-4

**Published:** 2023-04-15

**Authors:** Yurie Obata, Tomomi Yamada, Koichi Akiyama, Teiji Sawa

**Affiliations:** 1 Department of Anesthesiology, Yodogawa Christian Hospital, 1-7-50 Kunijima, Higashiyodogawaku, 533-0024 Osaka Japan; 2 Department of Anesthesiology, Kyoto Prefectural University of Medicine, Kyoto, Japan; 3 Department of Anesthesiology, Kindai University, Osaka, Japan

**Keywords:** Depth of anesthesia, Electroencephalogram, Hilbert–Huang transform

## Abstract

**Background:**

Anesthesiologists are required to maintain an optimal depth of anesthesia during general anesthesia, and several electroencephalogram (EEG) processing methods have been developed and approved for clinical use to evaluate anesthesia depth. Recently, the Hilbert–Huang transform (HHT) was introduced to analyze nonlinear and nonstationary data. In this study, we assessed whether the changes in EEG characteristics during general anesthesia that are analyzed by the HHT are useful for monitoring the depth of anesthesia.

**Methods:**

This retrospective observational study enrolled patients who underwent propofol anesthesia. Raw EEG signals were obtained from a monitor through a previously developed software application. We developed an HHT analyzer to decompose the EEG signal into six intrinsic mode functions (IMFs) and estimated the instantaneous frequencies (HHT_IF) for each IMF. Changes over time in the raw EEG waves and parameters such as HHT_IF, BIS, spectral edge frequency 95 (SEF95), and electromyogram parameter (EMGlow) were assessed, and a Gaussian process regression model was created to assess the association between BIS and HHT_IF.

**Results:**

We analyzed EEG signals from 30 patients. The beta oscillation frequency range (13–25 Hz) was detected in IMF1 and IMF2 during the awake state, then after loss of consciousness, the frequency decreased and alpha oscillation (8–12 Hz) was detected in IMF2. At the emergence phase, the frequency increased and beta oscillations were detected in IMF1, IMF2, and IMF3. BIS and EMGlow changed significantly during the induction and emergence phases, whereas SEF95 showed a wide variability and no significant changes during the induction phase. The root mean square error between the observed BIS values and the values predicted by a Gaussian process regression model ranged from 4.69 to 9.68.

**Conclusions:**

We applied the HHT to EEG analyses during propofol anesthesia. The instantaneous frequency in IMF1 and IMF2 identified changes in EEG characteristics during induction and emergence from general anesthesia. Moreover, the HHT_IF in IMF2 showed strong associations with BIS and was suitable for depicting the alpha oscillation. Our study suggests that the HHT is useful for monitoring the depth of anesthesia.

**Supplementary Information:**

The online version contains supplementary material available at 10.1186/s12871-023-02082-4.

## Background

In general anesthesia management, too shallow general anesthesia leads to a risk of intraoperative arousal, while excessively deep anesthesia may be associated with delayed arousal and postoperative cognitive dysfunction [1]. Therefore, anesthesiologists would like to maintain an optimal anesthesia level by evaluating the depth of anesthesia.

Because differences in the dosage of general anesthetics affect electroencephalogram (EEG) recordings, capturing the changes in EEG during general anesthesia is considered a practical method for monitoring the depth of anesthesia. Currently, several processing methods for measuring EEG changes have been developed and approved for clinical use in depth-of-anesthesia monitoring [2]. The Bispectral Index (BIS™, Medtronic, Boulder, CO, USA) based on frequency domain analysis is the most-used method to date [3]. Other examples include the Patient State Index (PSI, Masimo Corp., Irvine, CA, USA) obtained from EEG power, frequency, and phase information [4], M-entropy (GE Healthcare, Helsinki, Finland), which measures state entropy [5], and the auditory evoked potential (AEP) that measures the latency of a cortical response to auditory stimuli [6]. More recently, analysis methods using algorithms other than Fourier analysis have been reported. Mode decomposition, which can extract essential characteristic structural information hidden in multidimensional time series data, is one such new method. Mode decomposition extracts characteristic unit components, “modes”, that make up a phenomenon in the data [7]. The waveform is decomposed into characteristic modes, also called intrinsic mode functions (IMFs), and when all modes are added together they reproduce the original waveform. When mode decomposition is applied to EEG analysis, the resulting mode-decomposed EEG waveforms are EEGs with characteristics in a specific narrow frequency band. Among the various mode decomposition methods described, empirical mode decomposition (EMD) is a time-frequency analysis method that was proposed by Huang et al. in 1998 [8]. Analysis using EMD was later coupled with the Hilbert transform and defined as the Hilbert–Huang transform (HHT). Several trials utilizing the HHT to evaluate EEG changes during general anesthesia have recently been reported [9–11]. However, none of these studies showed the dynamic changes of the instantaneous frequencies in each IMF throughout the anesthesia. We hypothesized that one of these IMFs identifies the changes in the EEG signal and it represents the action of anesthesia. In this study, we analyzed the changes in EEG during the induction, maintenance, and emergence of general anesthesia under intravenous propofol as time-series changes in the instantaneous frequency characteristics of the HHT, and investigated whether these HHT characteristics caused by anesthetic administration are useful for monitoring the depth of anesthesia.

## Methods

All experiment protocols involving humans were conducted in accordance with the principles of the Declaration of Helsinki. The current study was approved by the Institutional Review Board (IRB) for Human Experiments at the Kyoto Prefectural University of Medicine (KPUM) (No. ERB-C-1074-2), and the IRB of Yodogawa Christian Hospital (YCH) (No. 2020-023). For this non-interventional and noninvasive retrospective observational study, the requirement for informed patient consent was waived by the IRB of KPUM; patients were provided with an opt-out option, about which they were notified in the preoperative anesthesia clinic. However, written informed consent was obtained from patients involved in the study at YCH. Patients who underwent general anesthesia with propofol for anesthetic induction and maintenance were enrolled. Patients who had medical contraindications to propofol, who underwent brain surgery or cardiac surgery, and those aged 75 years or older were excluded, as were emergency cases.

## Anesthesia management

In our facility, the use of a BIS monitor is routine for adult patients who undergo surgery involving general anesthesia. The anesthesiologists in charge of management did not receive notice of the study and planned the anesthesia methods for scheduled surgeries following our facility’s standard care protocol, without any feedback regarding the online analysis of processed EEG signals. Patients were not premedicated before anesthesia induction, in accordance with our facility’s standard protocol.

A BIS electrode sensor was attached to the forehead before the induction of anesthesia. Anesthesia was induced with a target-controlled intravenous infusion of propofol (1.5–2.0 mg·kg^− 1^, 1% Diprivan injection kit, Sandoz K.K., Tokyo, Japan) to provide an effect-site concentration of 3.0–4.0 µg·mL^− 1^, and a continuous intravenous infusion of remifentanil (0.3–0.5 µg·kg^− 1^ min^− 1^). After there was no response to calling and disappearance of the eyelash reflex was confirmed, rocuronium (0.8–1.0 mg·kg^− 1^) was intravenously administered and tracheal intubation was conducted. The anesthesia was maintained with propofol at an effect-site concentration of 2.0–3.0 µg·mL^− 1^, small doses of fentanyl (1–2 µg·kg^− 1^ per dose), continuous intravenous infusion of remifentanil (0.125–0.25 µg·kg^− 1^·minute^− 1^), and additional maintenance doses of rocuronium (0.2 mg·kg^− 1^ at intervals of 20–30 min). At the phase of emergence from general anesthesia, sugammadex (4 mg·kg^− 1^) was administered to reverse the neuromuscular blockade effect. Patients were extubated when they were able to follow commands and showed spontaneous ventilation.

## Data acquisition

We previously developed a software application named “EEG Analyzer” to obtain raw EEG signals from a BIS A-2000 monitor (version 3.23; Medtronic, Minneapolis, MN, USA) through an RS-232 interface [12]. A BIS Quatro sensor was attached on the frontal region, in accordance with the manufacturer’s recommendations and single frontal channel EEG was recorded. The digitized EEG packets with a sampling frequency of 128 Hz were obtained through the serial output of the BIS monitor that sent a packet of sixteen sets of EEG mV data (32 bits) and eight packets per second (128 Hz). The EEG signals were saved on a personal computer (PC) as a tsv file. The obtained EEG signal was already preprocessed by the BIS monitor. The details of the signal preprocessing were described previously [13]. We didn’t apply any further preprocessing. The BIS value, spectral edge frequency 95 (SEF95), suppression ratio (SR), and electromyogram (EMG) parameter EMGlow were also saved to the PC every 3 s. In this study, we analyzed the temporal changes in the associations between the raw EEG waves and parameters such as observed BIS, SEF95, and EMGlow. We focused on three 7-minute phases of anesthesia induction, anesthesia maintenance (30 min after the incision), and emergence from anesthesia.

## Hilbert–Huang transform

**Fig. 1:**
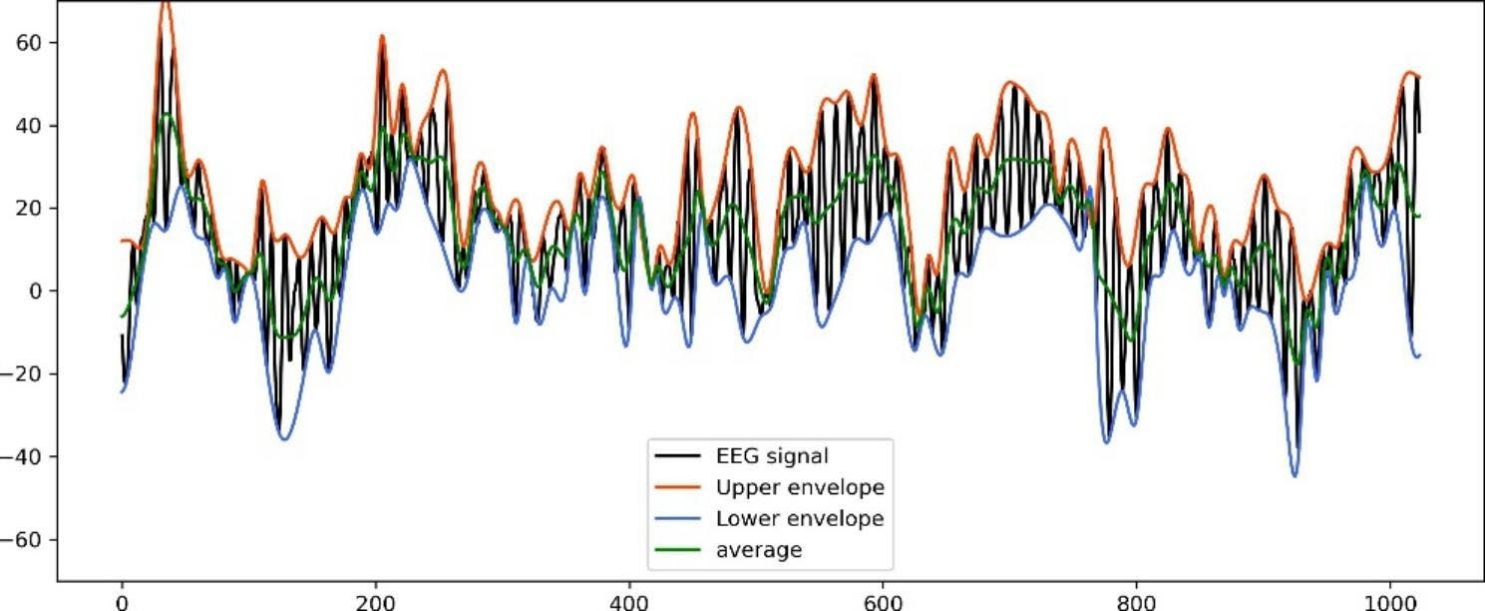
An EEG signal and its mean with the upper and lower envelopes This EEG signal was obtained from a 28-year-old woman during the anesthesia maintenance phase. The length and the sampling rate were 8 s and 128 Hz, respectively

For an EEG signal *x*(*t*), the EMD decomposes the signal into a series of intrinsic mode functions (IMFs), C*n* (*n* = 1, 2,. . ., N), where N is the number of IMFs. EMD starts by identifying the upper and lower envelopes of *x*(*t*); then, the mean of the upper and lower envelopes is designated as (*m*_1_) (Fig. 1, the program code is shown in Supplementary Digital Document 1). The difference between *x*(*t*) and *m*_1_ is the first component, *h*_1_.

1 $$x(t) - {m_1} = {h_1}$$

Ideally, *h*_1_ should be an IMF. If *h*_1_ does not satisfy all requirements of an IMF, the sifting process is repeated. The IMFs need to meet the following two requirements: (1) each IMF has the same number of zero crossings and extremes; and (2) the IMF is symmetric with respect to the local mean. The IMFs were calculated using the processing function “void emd(< double > eeg_data)”. After EMD, a signal *x*(*t*) can be written as

2 $$x(t) = \sum\limits_{i = 1}^{n - 1} {imf{{(t)}_i} + {r_n}(t)}$$

The Hilbert transform is applied to the IMF components and the analytic signal Z(t) is obtained as follows [10]:

3 $$Z(t) = imf(t) + iH[imf(t)] = a(t){e^{i\smallint \omega (t)dt}}$$

in which

4 $$a(t) = \sqrt {im{f^2}(t) + {H^2}[imf(t)]}$$

5 $$\omega (t) = \frac{d}{{dt}}\left[ {\arctan (H[imf(t)]/imf(t))} \right]$$

6 $$h(\omega ) = \int {H(\omega ,t)dt}$$

where *ω*(*t*) and *a*(*t*) are the instantaneous frequency and amplitude of the IMF used to obtain a time-frequency distribution for signal *x*(*t*) and the Hilbert amplitude spectrum *H*(*ω*, *t*). In order to use the unique definition of instantaneous frequency, we need to decompose a complex data set into IMF components so that an instantaneous frequency can be assigned to each IMF. Otherwise, the instantaneous frequencies will be non-physical negative frequency values which are meaningless [8].

**Fig. 2:**
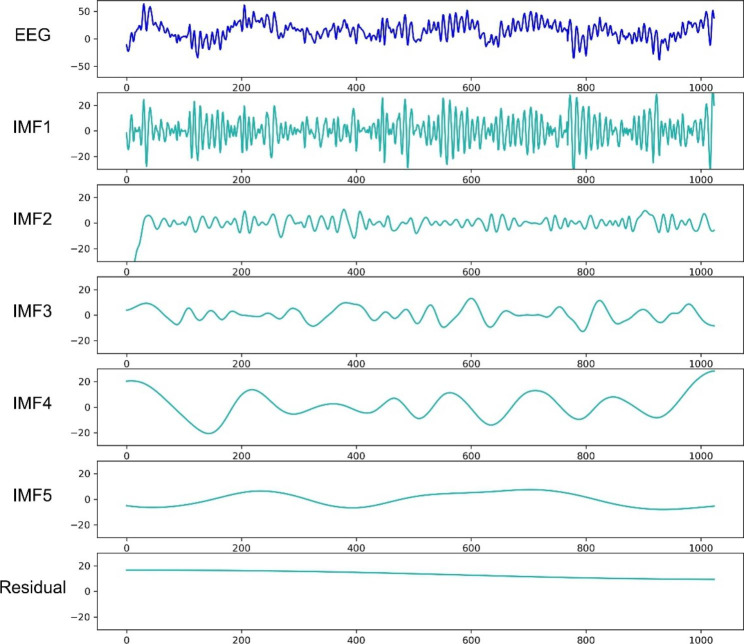
A representative example of empirical mode decomposition. The EEG signal is the same as that in Fig. 1. The signal is decomposed into five IMFs and a residual

An 8-s epoch of an EEG series from a patient in the anesthesia maintenance state and the EMD of this EEG epoch are shown in Fig. 2. (The program code is shown in Supplementary Digital Document 1.) This EEG series is composed of five IMFs and a residual. These IMFs are near-orthogonal components that have no overlap in frequency. The summation of the IMFs and the residual is equivalent to the original EEG series, according to Eq. (2).

**Fig. 3:**
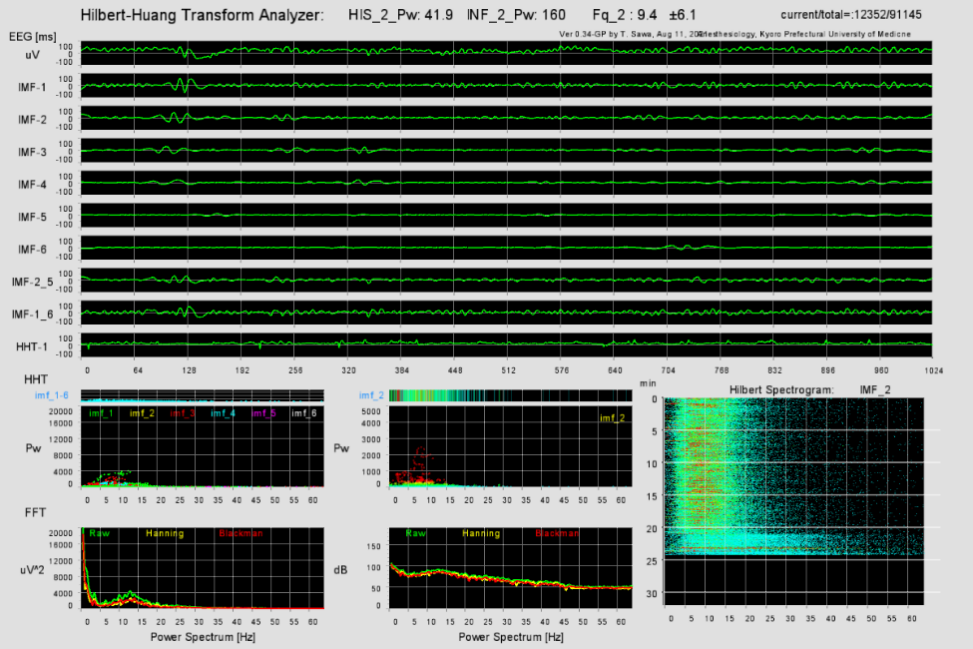
HHT analyzer. This figure shows the graphical user interface of the HHT analyzer. The upper half of the window shows the original EEG signal and its IMFs. The lower half windows show the power spectrums analyzed using a fast Fourier transform (left) and a Hilbert–Huang transform (middle). In addition, the Hilbert spectrogram is shown in the lower right

We developed the HHT analyzer to perform the HHT and display the Hilbert spectrogram using Processing ver. 3.5.4 (Processing Foundation) [14]. First, the program performs EMD to decompose the waveform into six IMFs. Then, the Hilbert transform is applied to each IMF to obtain instantaneous frequencies and amplitudes. We used IntelliJ IDEA ver. 2021.2 (JetBrains, Praha, Czech Republic) to handle two Java jar files named emd.jar and jdsp.jar. The former was downloaded from an open-access website [15] and the latter was collected from JDSP ver. 0.7.0, which is a java library for digital signal processing [16]. These components were integrated using the integrated development environment of Processing ver. 3.5.4, and a graphical user interface (GUI) for the Hilbert spectrogram was constructed (Fig. 3 and Supplementary Video File 1). The EEG waveform, IMFs, Fourier power spectrum, and Hilbert power spectrum were also displayed on the GUI. The HHT analysis was performed, and the GUI was updated every 8 s. The instantaneous frequencies, as well as the amplitudes, were automatically saved to the PC.

### Statistical analysis

Continuous variables are reported as mean ± standard deviation (SD) or median and interquartile range (IQR), and categorical variables as proportions. All EEG-related data were obtained from our EEG analyzer and HHT analyzer as tsv files. The files were opened in Microsoft Excel (Microsoft Office 2019, Microsoft Corporation, Redmond, WA, USA) and saved as xlsx files. The files were then transferred to GraphPad Prism vision 9.4.1 (GraphPad Software, San Diego, CA, USA) to perform part of the statistical analysis. The Wilcoxon matched-pairs signed rank test was used to compare changes in various EEG parameters between the first and last time points of the induction, maintenance, and emergence phases.

Gaussian process regression (GPR), supported by the Gaussian framework GPy (ver. 1.9.8) [17] in the Python programming language (ver. 3.6), was applied for the regression analysis between BIS and HHT_IF. GPR was performed in Python’s Anaconda Navigator (ver. 1.9.7, Anaconda, Inc., Austin, TX, USA) using the Jupyter Notebook environment (ver. 1.0.0, Project Jupyter; sample program code is shown in Supplementary Digital Document 2: the Jupyter Notebook Python code for GPR with a combination of Periodic Exponential kernel and Matern 32 kernel to determine the relationship between HHT_IF and BIS measured using Python’s GPy module). The regression curve was created using the posterior predictive distribution of the mean values obtained from 20 patients, and the quality of the curve fitting was assessed using the data obtained from the other ten patients. Root mean square error (RMSE) and coefficients of determination (*R*^2^) were calculated as estimators of the regression analyses. P < 0.05 was considered statistically significant.

## Results

We collected and analyzed EEG data from 30 patients. The patients’ demographics and clinical characteristics are summarized in Table 1. No patients had an atrial pacemaker or any known cerebrovascular disease. A forced-air-warming blanket was not placed on the forehead and the body temperature was maintained within the normal range in all patients.

**Table 1:** Demographic data and clinical characteristics of the patients

Variables	n = 30
Age [y.o.]	45 (36, 56)
Gender	Male: 9 (30%); Female: 21 (70%)
Height [cm]	161 (156, 168)
Weight [kg]	58 (51, 68)
BMI [kg/m2]	22.0 (20.0, 25.2)
ASA-PS	1: 17 (56.7%); 2: 12 (40.0%); 3: 1(3.3%)
Comorbidities	
Hypertension	5 (16.7%)
Bronchial asthma	3 (10.0%)
Rheumatoid arthritis	1 (3.3%)
None	21 (70.0%)
Duration of anesthesia [min]	158 (112, 205)
Duration of surgery [min]	97 (60, 144)
Surgical procedures	
Laparoscopic abdominal surgery	13 (43.3%)
Breast surgery	5 (16.7%)
Bone and joint surgery	5 (16.7%)
Transurethral ureterolithotripsy	3 (10.0%)
Otorhinolaryngological surgery	2 (6.7%)
Others	2 (6.7%)

Data are expressed as median (interquartile range) or counts (percentage).

BMI: body mass index; ASA-PS: American Society of Anesthesiologists physical status.

**Fig. 4:**
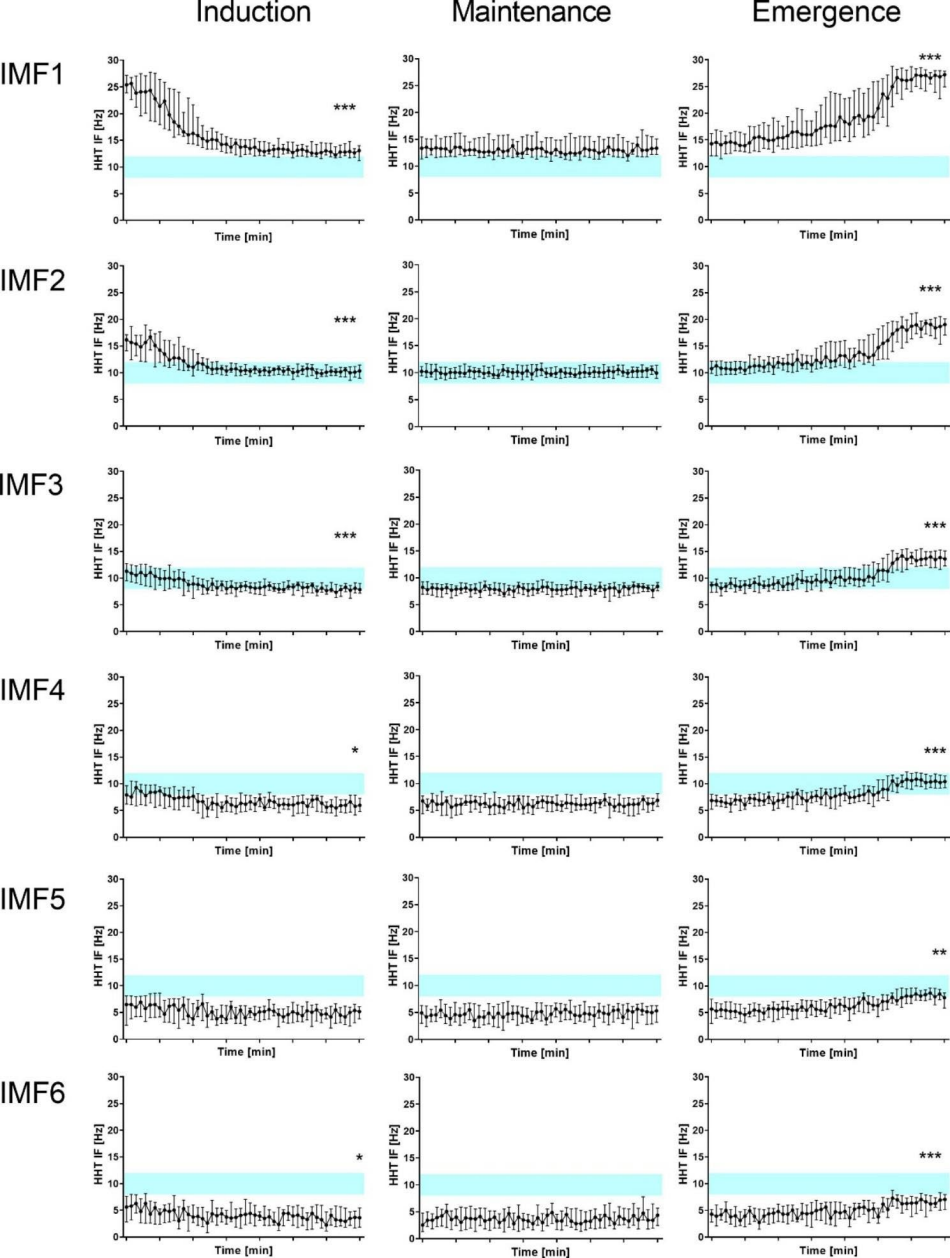
Instantaneous frequencies during induction, maintenance, and emergence phases. The EEG signals of 30 patients were analyzed, and the instantaneous frequencies (HHT_IF) of each IMF were calculated. Each dot and error bar indicate the median and interquartile range of the HHT_IF. The region shaded in light blue indicates the frequency range of 8–12 Hz (alpha oscillations). *: p < 0.05; **: p < 0.01; ***: p < 0.001

The time-course changes in the instantaneous frequency in the induction, maintenance, and emergence phases obtained from IMF1, IMF2, IMF3, IMF4, IMF5, and IMF6 are shown in Fig. 4. Additionally, the median and IQR of the instantaneous frequencies in IMF1 to IMF6 at the first and last time points of the three phases are shown in Table 2. The highest frequency components were extracted in IMF1, with the frequency then being lower in order from IMF1 to IMF6. At the induction phase, just before the propofol was administered, the frequency range of beta oscillations (13–25 Hz) was detected in IMF1 and IMF2, and the frequency range of theta oscillations (4–7 Hz) was detected in IMF4, IMF5, and IMF6. Then, after loss of consciousness, the frequency range of alpha oscillations (8–12 Hz) was detected in IMF2, and continued to be detected in the maintenance phase. The frequency decreased from 25.4 (IQR 23.9, 27.2) Hz to 13.1 (11.2, 14.0) Hz in IMF1 (p < 0.001), from 16.2 (14.1, 17.1) Hz to 10.3 (9.0, 11.5) Hz in IMF2 (p < 0.001), from 11.3 (9.5, 12.8) Hz to 7.9 (7.2, 9.0) Hz in IMF3 (p < 0.001), from 7.9 (6.2, 9.6) Hz to 6.0 (4.8, 7.3) Hz in IMF4 (p = 0.02), and from 5.6 (2.8, 7.6) Hz to 3.6 (1.8, 5.5) Hz in IMF6 (p = 0.05). No significant changes in frequency were observed in IMF5 (p = 0.11).

**Table 2:** The instantaneous frequencies in IMF1 to IMF6 at the first and last time points of the three phases

						[Hz]
IMF No.	Induction		Maintenance		Emergence	
	First time point	Last time point	First time point	Last time point	First time point	Last time point
IMF1	25.4(23.9, 27.2)	13.1(11.2,14.0)***	13.3(11.4, 15.4)	13.4(12.2, 15.1)	14.3(12.0, 16.2)	27.2(24.9, 27.9)***
IMF2	16.2(14.1, 17.1)	10.3(9.0, 11.5)***	10.2(9.4, 11.2)	9.9(9.0, 11.4)	10.8(9.9, 12.2)	19.0(17.1, 20.1)***
IMF3	11.3(9.5, 12.8)	7.9(7.2, 9.0)***	8.2(7.0, 9.3)	8.4(7.6, 9.1)	8.7(7.4, 9.2)	13.6(12.3, 15.1)***
IMF4	7.9(6.2, 9.6)	6.0(4.8, 7.3)*	6.7(4.4, 7.7)	6.9(5.7, 8.1)	6.9(5.2, 8.0)	10.4(9.3, 11.5)***
IMF5	6.5(2.6, 8.1)	5.2(3.8, 6.1)	4.9(3.0, 6.3)	5.3(3.2, 6.2)	5.7(3.0, 7.5)	7.8(5.9, 8.7)**
IMF6	5.6(2.8, 7.6)	3.6(1.8, 5.5)*	2.6(1.3, 4.7)	4.4(2.5, 5.7)	4.3(2.8, 5.2)	7.1(5.2, 8.0)***

Data are expressed as median (interquartile range). *: p < 0.05; **: p < 0.01; ***: p < 0.001

At the emergence phase, the frequency increased from 14.3 (12.0, 16.2) Hz to 27.2 (24.9, 27.9) Hz in IMF1 (p < 0.001), from 10.8 (9.9, 12.2) Hz to 19.0 (17.1, 20.1) Hz in IMF2 (p < 0.001), from 8.7 (7.4, 9.2) Hz to 13.6 (12.3, 15.1) Hz in IMF3 (p < 0.001), from 6.9 (5.2, 8.0) Hz to 10.4 (9.3, 11.5) Hz in IMF4 (p < 0.001), from 5.7 (3.0, 7.5) Hz to 7.8 (5.9, 8.7) Hz in IMF5 (p = 0.002), and from 4.3 (2.8, 5.2) Hz to 7.1 (5.2, 8.0) Hz in IMF6 (p < 0.001). The frequency range of beta oscillations was seen in IMF1, IMF2, and IMF3.

**Fig. 5:**
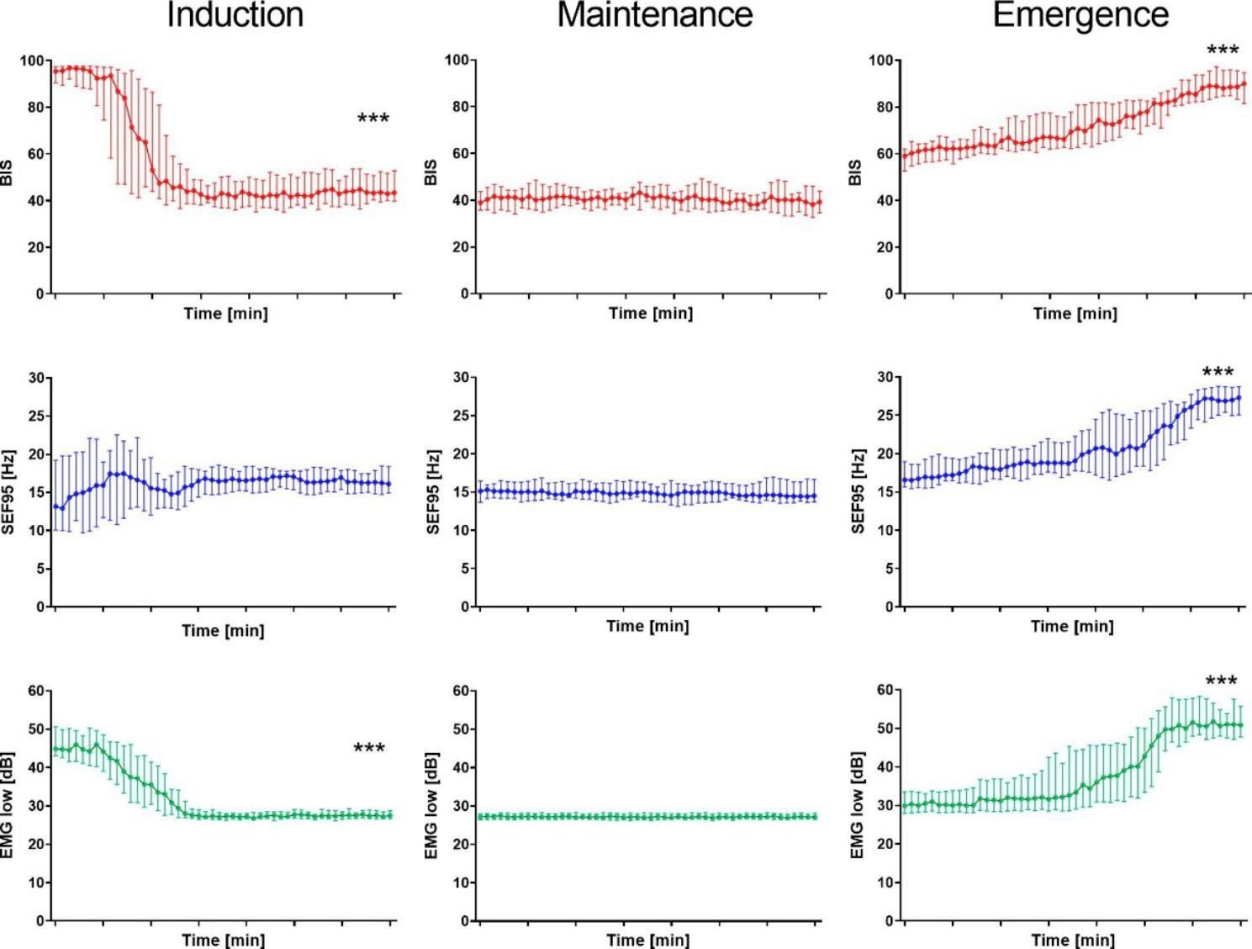
BIS, SEF95, and EMGlow during induction, maintenance, and emergence phases. The dots and error bars indicate the median and interquartile ranges of BIS (red), SEF95 (blue), and EMGlow (green). ***: p < 0.001

Figure 5 shows the time-course changes in BIS, SEF95, and EMGlow at the three phases. BIS decreased from 95 (90, 97) to 43 (40, 53) over the induction phase (p < 0.001), was maintained at around 40 during the maintenance phase, and increased from 59 (53, 62) to 90 (81, 95) in the emergence phase (p < 0.001). SEF95 showed a wide variability and no significant changes in the induction phase (p = 0.14), then increased from 16.6 (15.7, 19.0) Hz to 27.3 (25.0, 28.7) Hz in the emergence phase (p < 0.001). EMGlow decreased from 44.9 (43.1, 50.6) dB to 27.5 (26.7, 28.7) dB in the induction phase (p < 0.001), and increased from 29.9 (28.0, 33.5) dB to 50.8 (47.7, 55.7) dB in the emergence phase (p < 0.001).

**Fig. 6:**
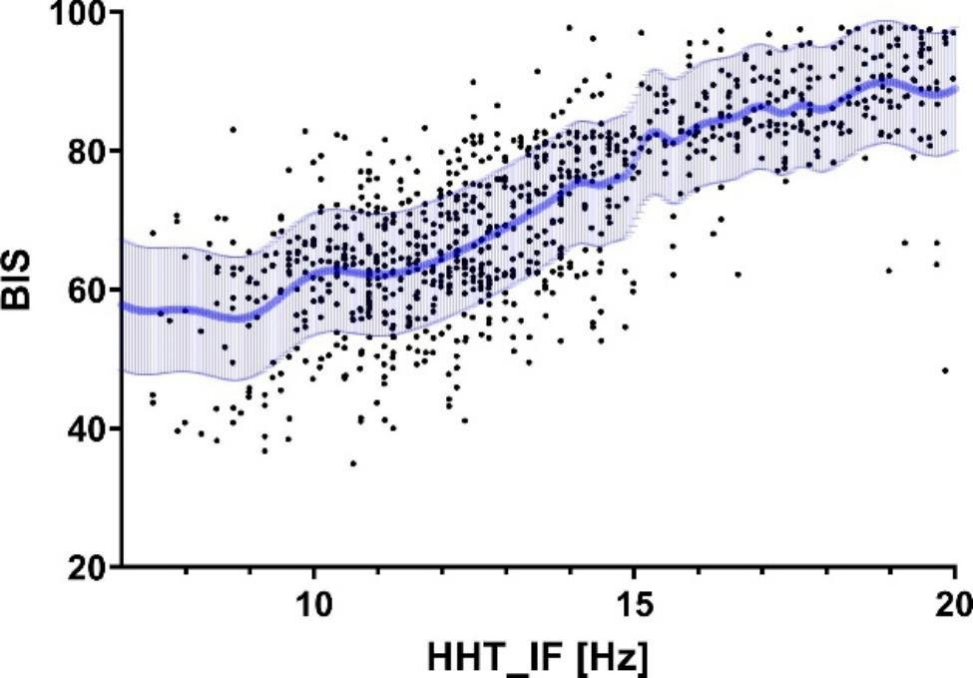
Relationship between BIS and HHT_IF. Each of the 20 patients had 50 data points during emergence from anesthesia and each data point is indicated by a dot, giving a total of 1000 points. The predicted mean and 68% credible interval for Gaussian process regression are depicted in blue

**Fig. 7:**
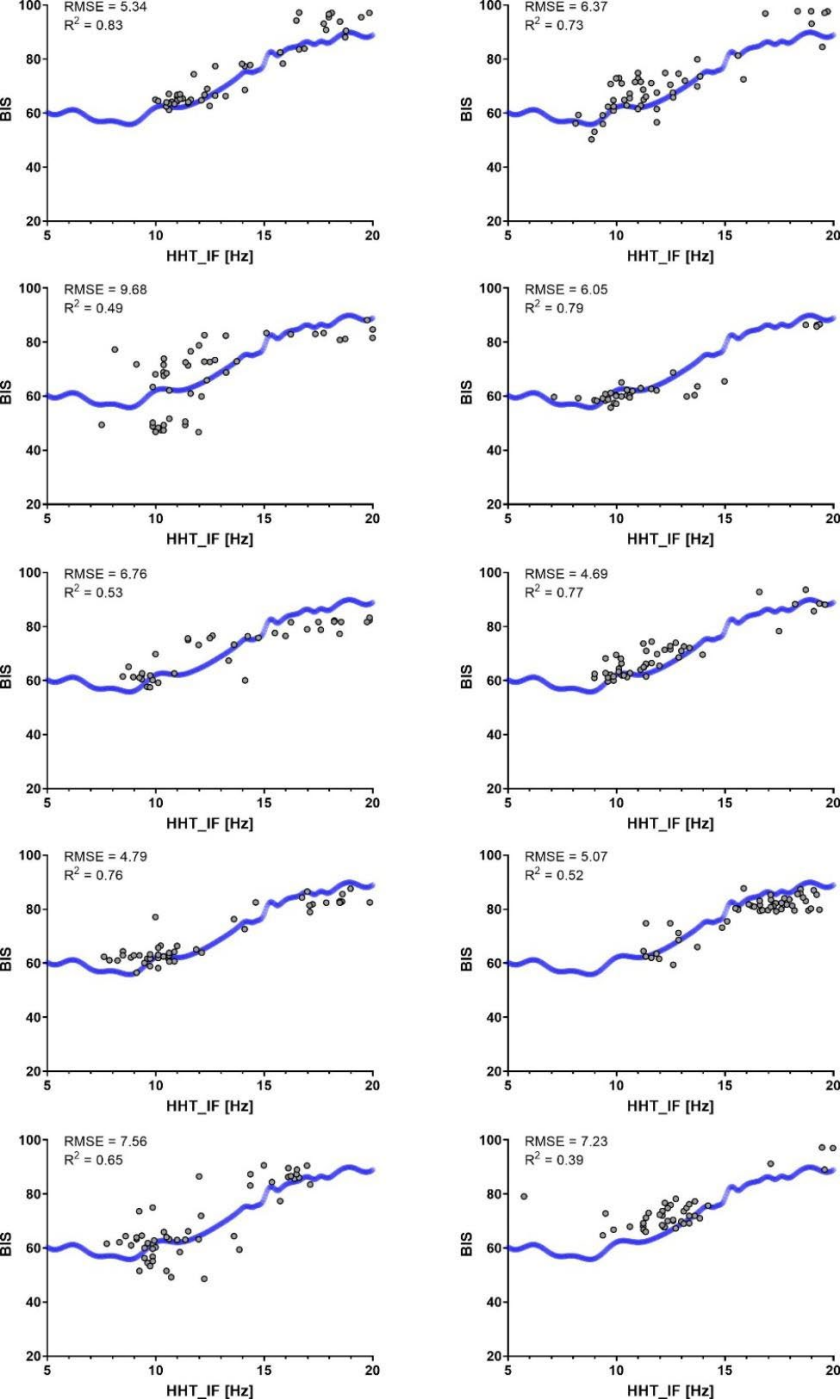
The quality of curve fitting for 10 individual patients. The regression line was obtained from the Gaussian process regression shown in Fig. 6. Each panel shows a scatter plot showing the relationship between BIS and HHT_IF in an individual patient. Root mean square error (RMSE) and coefficient of determination (R^2^) are shown in the upper left corners

According to the characteristics of the IMFs, we chose the instantaneous frequencies in IMF2 as candidates for GPR. The relationship between BIS and HHT_IF in the emergence phase is shown in Fig. 6. This GPR model was created using the data obtained from 20 patients. Figure 7 shows the quality of the curve fitting assessed using the data obtained from the other 10 patients. RMSE and *R*^2^ ranged from 4.69 to 9.68 and 0.39 to 0.83, respectively.

## Discussion

In the present study, we introduced the HHT analyzer and applied it to analysis of EEG data acquired during propofol anesthesia. The most important finding of this study is that the HHT-derived instantaneous frequencies can identify changes in EEG signals during induction of and emergence from general anesthesia. Furthermore, the HHT_IF showed strong associations with the BIS value. Our results suggest that these IMFs extract the main characteristics of the EEG signals. It means that our HHT analyzer has the potential to be a monitor of the action of anesthesia.

Monitoring of anesthesia with the BIS involves an electroencephalographic monitor that displays a processed dimensionless number between 0 and 100 [18]. BIS has been extensively studied and is now widely accepted as an index of the depth of anesthesia [19]. However, BIS is showing just a ‘number’ and using proprietary algorithms. BIS monitoring assumes that the same BIS value defines the same level of unconsciousness for all anesthetics, and the BIS value itself does not indicate any electroencephalographic information. On the other hand, raw EEG has limited use in the measurement of depth of anesthesia because the majority of clinicians do not have the time nor the skill to interpret the complexity of the raw EEG data [20]. Our study suggests that HHT analysis and the instantaneous frequencies in IMFs may help clinicians to interpret the EEG patterns which indicate the states such as wakefulness, light anesthesia, and deep anesthesia.

Although the complete algorithm used to estimate BIS value is a trade secret, the Fourier transform is involved in the algorithm to deconstruct the EEG signals into individual sine waves of differing amplitude and frequency [10]. The Fourier transform assumes that the signal is stationary within the short window periods. However, EEG is usually nonstationary, and the Fourier transform may not accurately exhibit the frequency band of the EEG signals. Moreover, a major limitation of the Fourier transform is the trade-off relationship between time and frequency resolutions [11]. This might be one of the reasons why a pure Fourier transform fails to assess the depth of anesthesia. In fact, our study shows that SEF95, which is a parameter directly derived from the power spectrum of the Fourier transform, does not identify the change in EEG during induction of anesthesia. In contrast, the BIS and the HHT_IF do demonstrate the change in EEG during induction. Compared to the classic Fourier transform, the advantage of HHT is good time and frequency resolutions. Also, HHT can be applied nonlinear and nonstationary data without any assumptions that Fourier transform requires.

To overcome some of the limitations of the Fourier analysis, the multitaper method was introduced [21]. Purdon et al. recorded high-density EEGs in young subjects during gradual induction of and emergence from unconsciousness with propofol, and computed spectrograms using the multitaper method [22]. During induction, the median frequency decreased from 23.1 to 12.0 Hz, and during emergence, the median frequency increased from 11.8 to 21.9 Hz in the transition period after the return of consciousness. These changes in frequencies are similar but slightly lower than our results for the instantaneous frequencies in IMF1. This difference might reflect the characteristics of EMD, which decomposes the EEG signal into IMFs in order of high to low frequencies.

The HHT has been used for frequency analysis in various fields, including motion capture data, electricity demand spectral analysis, and financial data analysis [23–25]. Some studies have used the HHT to analyze EEG signals during general anesthesia. Compared to these studies, the novelty of our study is that we focused on the dynamic changes of the instantaneous frequencies in each IMF throughout the anesthesia. We chose the number of IMF to be six so that all spectral frequency bands are assigned to any IMF. The high frequency bands are extracted in IMF1 and IMF2. Whereas the theta and delta oscillations are extracted in IMF4, IMF5, and IMF6.

Liu et al. demonstrated HHT analysis of EEG spectrograms under propofol anesthesia [11]. They showed that the dominant power was located in the delta (1–4 Hz) and alpha (8–12 Hz) bands for the unconsciousness period, and below 4 Hz during the awake state. However, their result concerning the dominant power during the awake state is inconsistent with previous reports. The typical EEG pattern in the awake state involves low-amplitude and high-frequency waves characterized by beta and gamma oscillations [26]. Our results support the fact that IMF1 and IMF2 capture these beta oscillations during the awake state.

Shalbaf et al. introduced a novel index named the Hilbert–Huang weighted regional frequency (HHWRF) and compared it with BIS values during propofol sedation in a small number of volunteers [27]. They observed a high correlation between HHWRF and BIS. Their original index, HHWRF, includes the summation of all instantaneous frequencies and amplitudes in all IMFs. As an alternative, we focus on each IMF as an extracted feature of the EEG. IMF1 and IMF2 are suitable for detecting the change in EEG during induction and emergence from anesthesia, and IMF2 also extracts the alpha oscillation during unconsciousness.

From the physiological point of view, the EEG is showing the net summation of microscopic currents produced in the cortex. Millions of postsynaptic potentials are asynchronously firing all over the cortex, summing to create a complicated signal. Higher cortical function is usually associated with desynchronization as neurons act more independently. Whereas anesthesia is associated with increasing cortical synchrony [13]. The synchrony is influenced by neuronal circuit loops involving the interaction of cortical and subcortical structures [26]. EEG patterns also reflect this interconnection. We think that the HHT seems to be a process to understand the complex brain activities by decomposing the EEG signal into several IMFs.

Propofol acts mainly as a positive allosteric modulator of γ-aminobutyric acid type A receptors in the brain [26]. Under propofol anesthesia, EEG is characterized by frontal alpha oscillations. Recently, increasing attention has been paid to the frontal alpha power in the clinical setting. Shao et al. showed that lower frontal alpha power is associated with a higher propensity for burst suppression and a potentially higher risk of postoperative neurocognitive disorders [28]. However, no EEG monitor can focus on and pick out the alpha oscillation. Our study demonstrated the alpha oscillations during the maintenance phase in the form of IMF2, and this IMF might be valuable for assessing the depth of anesthesia through its depiction of the alpha oscillation.

Our study has several limitations. First, we analyzed EEG from only young and middle-aged patients. It is well known that the EEG amplitude, as well as the alpha power, decreases with aging [29]. Further study is needed to investigate how our HHT analyzer works in elderly patients. Second, all patients received not only propofol, but also remifentanil, fentanyl, and rocuronium. All anesthetic agents affect the EEG signals and BIS value [30]. However, these anesthetic agents are usually used together in the clinical setting.

## Conclusions

We developed the HHT analyzer and analyzed frontal EEG during propofol anesthesia. The HHT analyzer decomposes the EEG signal into six IMFs. The instantaneous frequencies in IMF1 and IMF2 identify the changes in EEG characteristics that occur during induction and emergence from general anesthesia. Furthermore, IMF2 is suitable for depicting the alpha oscillation. Our study suggests that the HHT is useful for monitoring the depth of anesthesia.

## Electronic supplementary material

Below is the link to the electronic supplementary material.

Supplementary Material 1

Supplementary Material 2

Supplementary Material 3

## Data Availability

The datasets are available from the corresponding author on reasonable request.

## List of abbreviations

EEG: electroencephalogram
HHT: Hilbert–Huang transform
BIS: bispectral index
IMF: intrinsic mode function
IF: instantaneous frequency
SEF95: spectral edge frequency 95
PSI: patient state index
AEP: auditory evoked potential
EMD: empirical mode decomposition
IRB: institutional review board
KPUM: Kyoto Prefectural University of Medicine
YCH: Yodogawa Christian Hospital
PC: personal computer
SR: suppression ratio
EMG: electromyogram
GUI: graphical user interface
SD: standard deviation
IQR: interquartile range
GPR: Gaussian process regression
RMSE: root mean square error
*R*^2^: coefficients of determination
BMI: body mass index
ASA-PS: American Society of Anesthesiologists physical status
HHWRF: Hilbert–Huang weighted regional frequency.

## References

[1] Chan MT, Cheng BC, Lee TM, Gin T (2013). BIS-guided anesthesia decreases postoperative Delirium and Cognitive decline. J Neurosurg Anesthesiol.

[2] Shander A, Lobel GP, Mathews DM (2018). Brain monitoring and the depth of anesthesia: another goldilocks dilemma. Anesth Analg.

[3] Bruhn J, Bouillon TW, Shafer SL (2000). Bispectral index (BIS) and burst suppression: revealing a part of the BIS algorithm. J Clin Monit Comput.

[4] Kh L, Yh K, Yj S, Mk O (2015). The patient State Index is well balanced for propofol sedation. Hippokratia.

[5] Gruenewald M, Zhou J, Schloemerkemper N, Meybohm P, Weiler N, Tonner PH, Scholz J, Bein B (2007). M-Entropy guidance vs standard practice during propofol-remifentanil anaesthesia: a randomised controlled trial. Anaesthesia.

[6] Kurita T, Doi M, Katoh T, Sano H, Sato S, Mantzaridis H, Kenny GN (2001). Auditory evoked potential index predicts the depth of Sedation and Movement in response to skin incision during Sevoflurane Anesthesia. Anesthesiology.

[7] Dragomiretskiy K, Zosso D (2014). Variational mode decomposition. IEEE Trans Signal Process.

[8] Huang NE, Shen Z, Long SR, Wu MC, Shih HH, Zheng Q, Yen N-C, Tung CC, Liu HH (1998). The empirical mode decomposition and the Hilbert spectrum for nonlinear and non-stationary time series analysis. Proc R Soc Lond A.

[9] Tsai FF, Fan SZ, Lin YS, Huang NE, Yeh JR (2016). Investigating power density and the degree of nonlinearity in intrinsic components of anesthesia EEG by the hilbert-huang transform: an example using ketamine and alfentanil. PLoS ONE.

[10] Li X, Li D, Liang Z, Voss LJ, Sleigh JW (2008). Analysis of depth of anesthesia with Hilbert-Huang spectral entropy. Clin Neurophysiol.

[11] Liu Q, Ma L, Fan SZ, Abbod MF, Ai Q, Chen K, Shieh JS (2018). Frontal EEG temporal and spectral dynamics similarity analysis between propofol and desflurane induced anesthesia using hilbert-huang transform. Biomed Res Int.

[12] Hayase K, Kainuma A, Akiyama K, Kinoshita M, Shibasaki M, Sawa T (2021). Poincaré plot area of Gamma-Band EEG as a measure of Emergence from Inhalational General Anesthesia. Front Physiol.

[13] Rampil I (1998). A primer for EEG signal processing in anesthesia. Anesthesiology.

[14] Welcome to Processing!. / Processing.org. https://processing.org/. Accessed 21 Jan 2023.

[15] Google Code Archive. - Long-term storage for Google Code Project Hosting. https://code.google.com/archive/p/realtime-emd/. Accessed 8 Jan 2023.

[16] JDSP - Digital Signal Processing in Java. https://jdsp.dev/. Accessed 21 Jan 2023.

[17] GPy by SheffieldML. http://sheffieldml.github.io/GPy/. Accessed 8 Jan 2023.

[18] Avidan MS, Zhang L, Burnside BA, Finkel KJ, Searleman AC, Selvidge JA (2008). Anesthesia awareness and the Bispectral Index. N Engl J Med.

[19] Lewis SR, Pritchard MW, Fawcett LJ, Punjasawadwong Y (2019). Bispectral index for improving intraoperative awareness and early postoperative recovery in adults. Cochrane Database of Systematic Reviews.

[20] Hajat Z, Ahmad N, Andrzejowski J (2017). The role and limitations of EEG-based depth of anaesthesia monitoring in theatres and intensive care. Anaesthesia.

[21] Sawa T, Yamada T, Obata Y (2022). Power spectrum and spectrogram of EEG analysis during general anesthesia: Python-based computer programming analysis. J Clin Monit Comput.

[22] Purdon PL, Pierce ET, Mukamel EA, Prerau MJ, Walsh JL, Wong KFK (2013). Electroencephalogram signatures of loss and recovery of consciousness from propofol. Proc Natl Acad Sci U S A.

[23] Dong R, Cai D, Ikuno S (2020). Motion capture data analysis in the instantaneous frequency-domain using Hilbert-Huang transform. Sensors.

[24] Luque J, Anguita D, Pérez F, Denda R (2020). Spectral analysis of electricity demand using Hilbert–Huang transform. Sensors.

[25] Huang NE, Wu ML, Qu W, Long SR, Shen SSP (2003). Applications of Hilbert-Huang transform to non-stationary financial time series analysis. Appl Stoch Models Bus Ind.

[26] Purdon PL, Sampson A, Pavone KJ, Brown EN (2015). Clinical Electroencephalography for Anesthesiologists Part I: background and basic signatures. Anesthesiology.

[27] Shalbaf R, Behnam H, Sleigh JW, Voss LJ (2012). Using the Hilbert-Huang transform to measure the electroencephalographic effect of propofol. Physiol Meas.

[28] Shao YR, Kahali P, Houle TT, Deng H, Colvin C, Dickerson BC (2020). Low frontal alpha power is associated with the propensity for burst suppression: an electroencephalogram phenotype for a “vulnerable brain. Anesth Analg.

[29] Purdon PL, Pavone KJ, Akeju O, Smith AC, Sampson AL, Lee J et al. The Ageing Brain: Age-dependent changes in the electroencephalogram during propofol and sevoflurane general anaesthesia.Br J Anaesth2015;i46–i57. 10.1093/bja/aev213 PMC4501918 26174300

[30] Dahaba AA (2005). Different conditions that could result in the bispectral index indicating an incorrect hypnotic state. Anesth Analg.

