# Supplementary material for: Time-trend analysis of the center frequency of the intrinsic mode function from the Hilbert–Huang transform of electroencephalography during general anesthesia: a retrospective observational study
Source: BMC Anesthesiol. 2023 Apr 15;23:125. doi: 10.1186/s12871-023-02082-4 (PMC10105429; doi:10.1186/s12871-023-02082-4)
Supplement: Supplementary file 2 — Supplementary Material 2 [file 12871_2023_2082_MOESM2_ESM.pdf]

In [1]:

```
import emd #Empirical Mode Decomposition
import numpy as np
from scipy import ndimage
import matplotlib.pyplot as plt
import matplotlib.patches as patches
import pandas as pd
```

In [2]:

```
#Import data from Excel file
df = pd.read_excel('8_eeg_run_incision8s.xlsx')
x_eeg = df[['eeg']].values.reshape(-1,)
```

In [3]:

```
#Check the type of the captured data
type(df), type(x_eeg) #pandas.core.frame.DataFrame, numpy.ndarray
```

Out[3]:

```
(pandas.core.frame.DataFrame, numpy.ndarray)
```

In [4]:

```
#Number of data acquired
len(x_eeg) #The number of data
```

Out[4]:

```
1024
```

In [5]:

```
# Run a mask sift
imf = emd.sift.mask_sift(x_eeg, max_imfs=11)
#Decomposition into Intrinsic Mode Function:IMF
```

In [6]:

```
emd.plotting.plot_imfs(imf[:1024, :], cmap=True, scale_y=True)
```

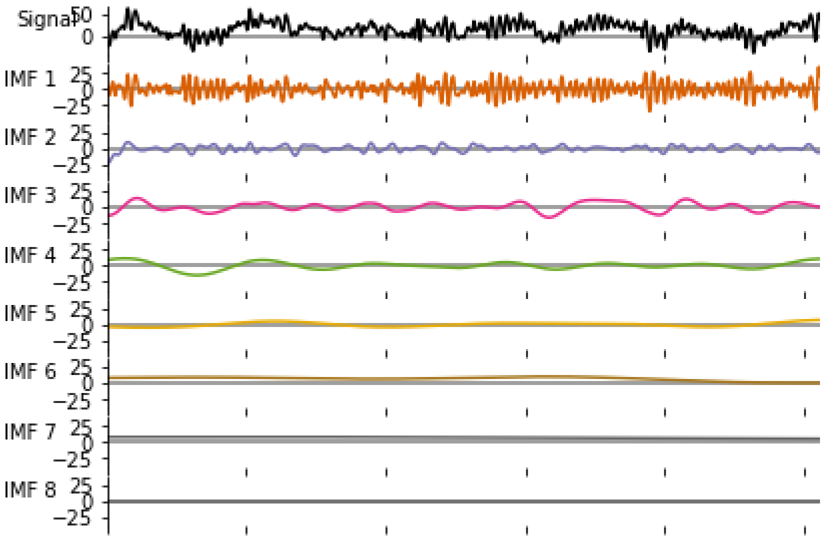

In [7]:

```
plt.plot(x_eeg)
```

Out[7]:

```
[<matplotlib.lines.Line2D at 0x7fc10210cac0>]
```

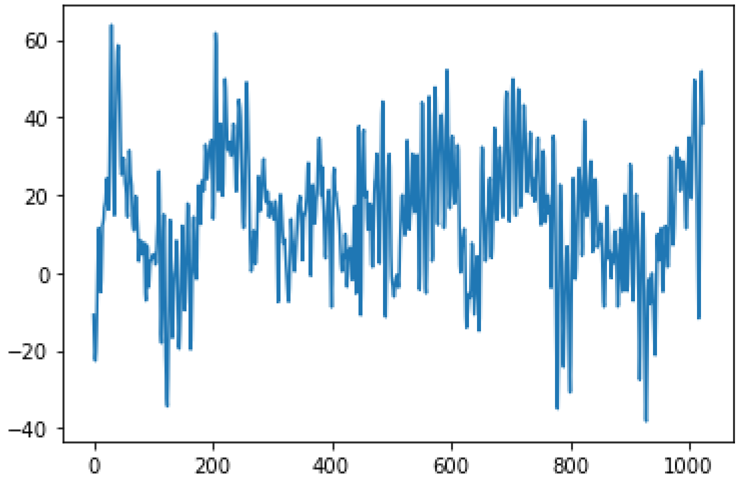

In [8]:

```
max_locs, max_mag = emd.sift.find_extrema(x_eeg)
min_locs, min_mag = emd.sift.find_extrema(x_eeg, ret_min=True)

plt.figure(figsize=(12, 3))
plt.plot(x_eeg, 'k')
plt.plot(max_locs, max_mag, 'or')
plt.plot(min_locs, min_mag, 'ob')
plt.legend(['Signal', 'Maxima', 'Minima'])
```

Out[8]:

```
<matplotlib.legend.Legend at 0x7fc10212acd0>
```

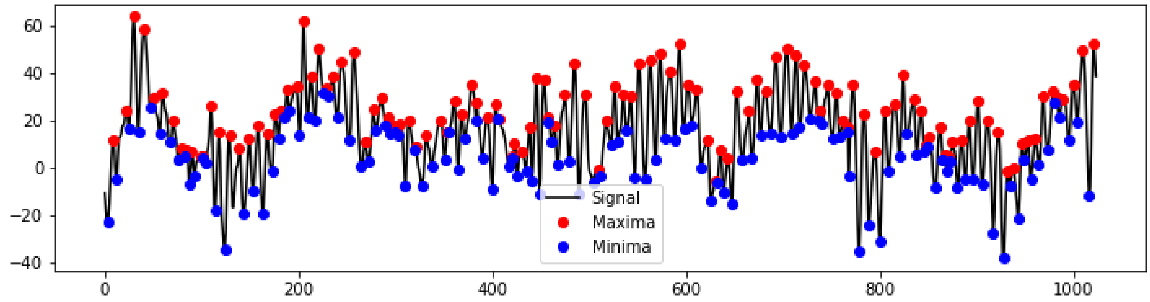

In [9]:

```
config = emd.sift.get_config('sift')
```

In [10]:

```
env_opts = config['envelope_opts']

upper_env = emd.utils.interp_envelope(x_eeg, mode='upper', **env_opts)
lower_env = emd.utils.interp_envelope(x_eeg, mode='lower', **env_opts)
avg_env = (upper_env+lower_env) / 2

plt.figure(figsize=(12, 5))
plt.subplot(111)
plt.plot(x_eeg, 'k')
plt.plot(upper_env, 'orangered')
plt.plot(lower_env, 'royalblue')
plt.plot(avg_env, 'green')
plt.ylim(-70, 70)
plt.legend(['EEG signal', 'Upper envelope', 'Lower envelope', 'average'])

plt.savefig("envelope.jpg", format="jpg", dpi=300)
```

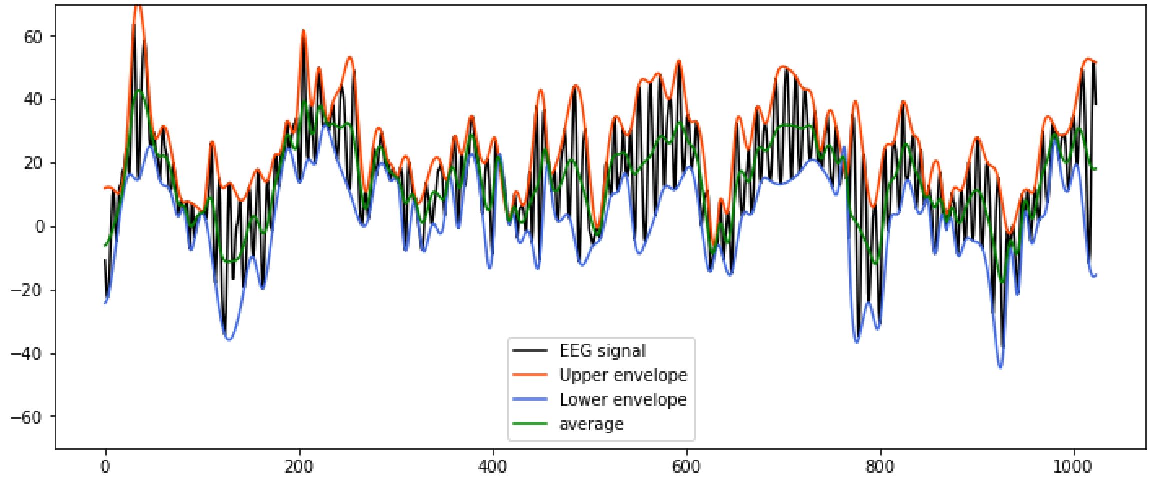

In [13]:

```
#Extract the options for get_next_imf
imf_opts = config['imf_opts']

imf1, continue_sift = emd.sift.get_next_imf(x_eeg[:, None],           **imf_opts)
imf2, continue_sift = emd.sift.get_next_imf(x_eeg[:, None]-imf1,      **imf_opts)
imf3, continue_sift = emd.sift.get_next_imf(x_eeg[:, None]-imf1-imf2, **imf_opts)
imf4, continue_sift = emd.sift.get_next_imf(x_eeg[:, None]-imf1-imf2-imf3, **imf_opts)
imf5, continue_sift = emd.sift.get_next_imf(x_eeg[:, None]-imf1-imf2-imf3-imf4, **imf_opts)

plt.figure(figsize=(12, 12))
plt.subplot(711)
plt.plot(x_eeg, 'k', color='blue')
plt.ylim(-70, 70)
plt.title('EEG', x=-0.1,y=0.4, fontname='Arial', fontsize='18')

plt.subplot(712)
plt.plot(imf1, 'k', color='lightseagreen')
plt.ylim(-30, 30)
plt.title('IMF1', x=-0.1,y=0.4, fontname='Arial', fontsize='18')

plt.subplot(713)
plt.plot(imf2, 'k', color='lightseagreen')
plt.ylim(-30, 30)
plt.title('IMF2', x=-0.1,y=0.4, fontname='Arial', fontsize='18')

plt.subplot(714)
plt.plot(imf3, 'k', color='lightseagreen')
plt.ylim(-30, 30)
plt.title('IMF3', x=-0.1,y=0.4, fontname='Arial', fontsize='18')

plt.subplot(715)
plt.plot(imf4, 'k', color='lightseagreen')
plt.ylim(-30, 30)
plt.title('IMF4', x=-0.1,y=0.4, fontname='Arial', fontsize='18')

plt.subplot(716)
plt.plot(imf5, 'k', color='lightseagreen')
plt.ylim(-30, 30)
plt.title('IMF5', x=-0.1,y=0.4, fontname='Arial', fontsize='18')

plt.subplot(717)
plt.plot(x_eeg[:, None]-imf1-imf2-imf3-imf4-imf5, 'k', color='lightseagreen')
plt.ylim(-30, 30)
plt.title('Residual', x=-0.1,y=0.4, fontname='Arial', fontsize='18')

plt.savefig("emd.jpg", format="jpg", dpi=300)
```

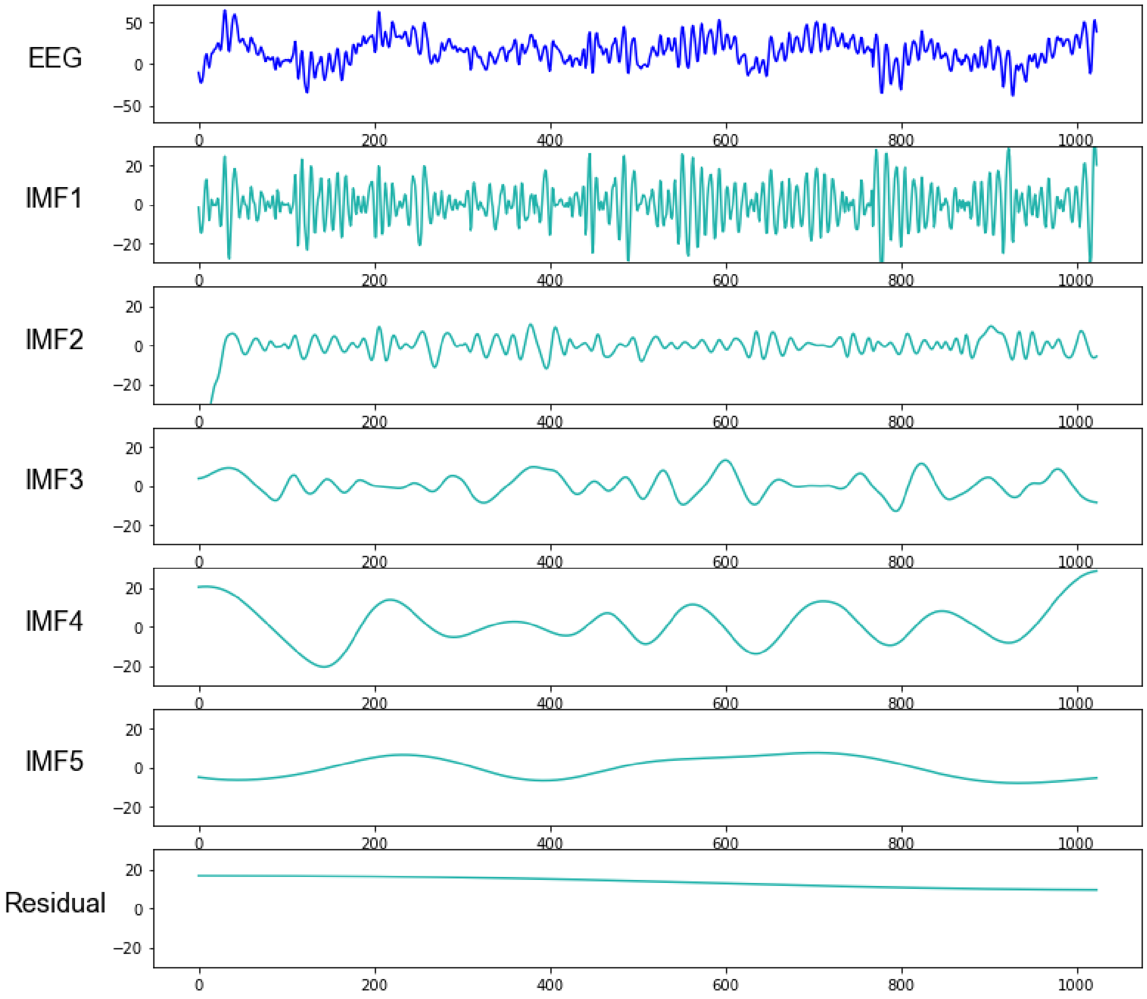
EEG
IMF1
IMF2
IMF3
IMF4
IMF5
Residual
50
0
−50
20
0
−20
0
200
400
600
800
1000

In [ ]:
