# Supplementary material for: Time-trend analysis of the center frequency of the intrinsic mode function from the Hilbert–Huang transform of electroencephalography during general anesthesia: a retrospective observational study
Source: BMC Anesthesiol. 2023 Apr 15;23:125. doi: 10.1186/s12871-023-02082-4 (PMC10105429; doi:10.1186/s12871-023-02082-4)
Supplement: Supplementary file 3 — Supplementary Material 3 [file 12871_2023_2082_MOESM3_ESM.pdf]

In [1]:

```
#from sklearn.gaussian_process import GaussianProcessRegressor
import GPy
import numpy as np
import matplotlib.pyplot as plt
%matplotlib inline
import pandas as pd
```

In [2]:

```
df = pd.read_csv('N30_IMF2_extubation_N20_10_3.csv')
```

In [3]:

```
x_pretrain = df[['HHT_IF']].values.reshape(-1,)
x_train = x_pretrain[~np.isnan(x_pretrain)]
y_pretrain = df[['BIS']].values.reshape(-1,)
y_train = y_pretrain[~np.isnan(y_pretrain)]
```

In [4]:

```
fig = plt.figure()
plt.scatter(x_train, y_train, marker=".", s=5, label="Data")
plt.xlim(10, 30)
plt.ylim(30, 100)
plt.legend()
plt.title("HHT extubation")
plt.xlabel('HHT_IF')
plt.ylabel('BIS')
plt.savefig("training_data_odd.png", dpi=150)
fig.savefig('extubation_HHT-IF_BIS_p0.pdf')
```

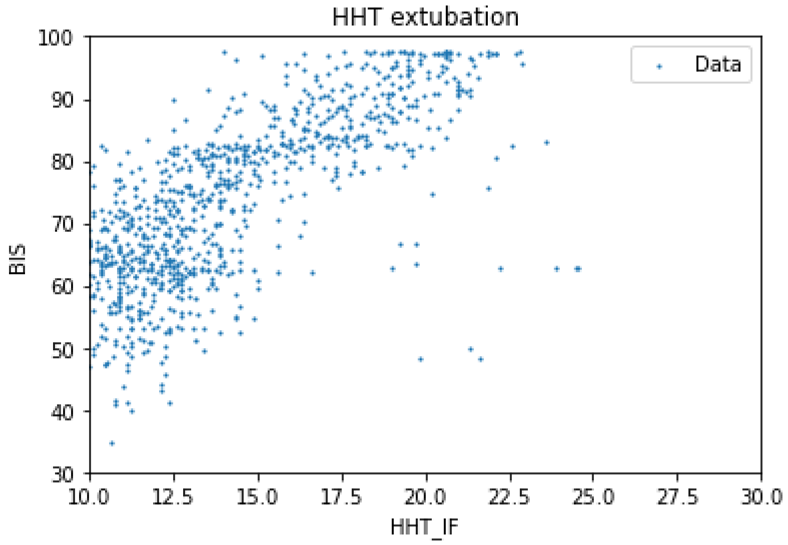

In [8]:

```
#kern = GPy.kern.RBF(input_dim=1)
kern = GPy.kern.PeriodicExponential(lengthscale=.1, variance=3) * GPy.kern.Matern32(1)
# kern = GPy.kern.RatQuad(1, lengthscale=.5, variance=.3)
# kern = GPy.kern.White(input_dim=1)
# kern = GPy.kern.RBF(input_dim=1) + GPy.kern.Bias(input_dim=1)
#kern = GPy.kern.PeriodicExponential(input_dim=1)

kern.plot()
plt.savefig("gpy_kern1.png", dpi=150)
```

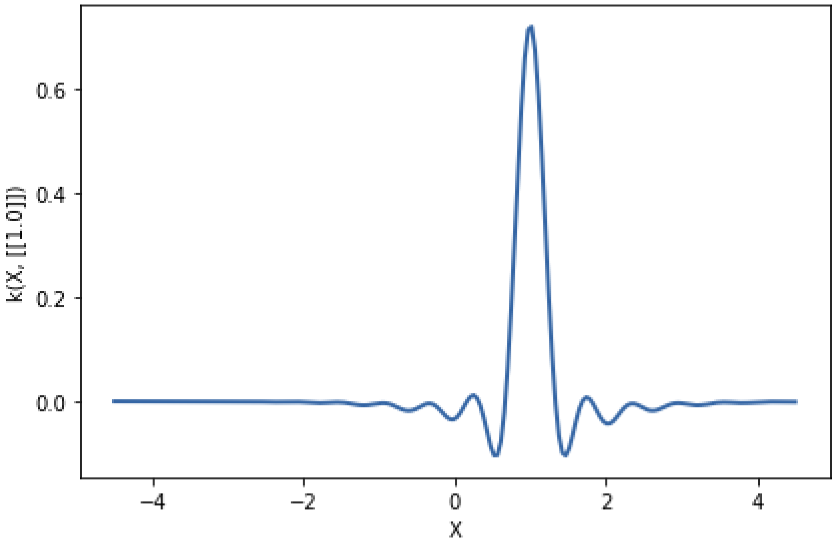

In [9]:

```
def plot_result(x_test, mean, std):
    plt.plot(x_test[:, 0], mean, color="C0", label="predict mean")
    plt.fill_between(x_test[:, 0], mean + std, mean - std, color="C0", alpha=.3,label= "1 sigma
confidence")
    plt.plot(x_train, y_train, ".",label= "training data", markersize=0.5)
```

In [25]:

```
import GPy.kern as gp_kern
kern = GPy.kern.PeriodicExponential(lengthscale=.1, variance=3) * GPy.kern.Matern32(1)
#kern = gp_kern.RBF(input_dim=1)
#kern = gp_kern.PeriodicMatern32(input_dim=1) * gp_kern.RBF(input_dim=1)
# kern = gp_kern.RBF(input_dim=1) + gp_kern.Bias(input_dim=1)
#kern = gp_kern.Linear(input_dim=2) * gp_kern.Matern52(input_dim=2)
#kern = gp_kern.PeriodicExponential(input_dim=1)

gpy_model = GPy.models.GPRegression(X=x_train.reshape(-1, 1),
                                    Y=y_train.reshape(-1, 1),
                                    kernel=kern, normalizer=None)
```

In [26]:

```
from IPython.display import display
display(gpy_model)
```

Model: GP regression
Objective: 264853.1976852516
Number of Parameters: 6
Number of Optimization Parameters: 6
Updates: True

GP_regression.	value	constraints	priors
mul.periodic_expone	3.0	+ve	
mul.periodic_expone	0.1	+ve	
mul.periodic_expone	6.283185307179586	+ve	
mul.Mat32.variance	1.0	+ve	
mul.Mat32.lengthsca	1.0	+ve	
Gaussian_noise.vari	1.0	+ve	

In [27]:

```
fig = plt.figure(figsize=(6,8))
ax1 = fig.add_subplot(211)
gpy_model.plot(ax=ax1, plot_density=True)  # Gpy model before optimization
gpy_model.optimize()
ax2 = fig.add_subplot(212, sharex=ax1)
gpy_model.plot(ax=ax2, plot_density=True)  # Gpy model after optimization
```

Out[27]:

```
{'dataplot': [<matplotlib.collections.PathCollection at 0x1e21b189dc0>],
 'gpmean': [[<matplotlib.lines.Line2D at 0x1e21b2a9220>]],
 'gpdensity': [[<matplotlib.collections.PolyCollection at 0x1e21b2a9430>]]}
```

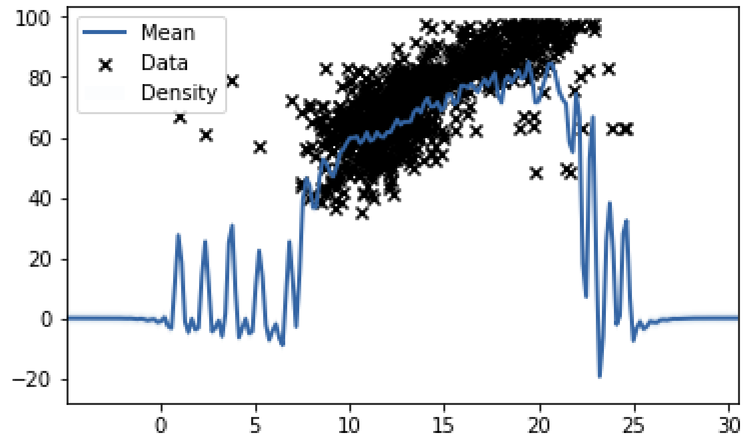

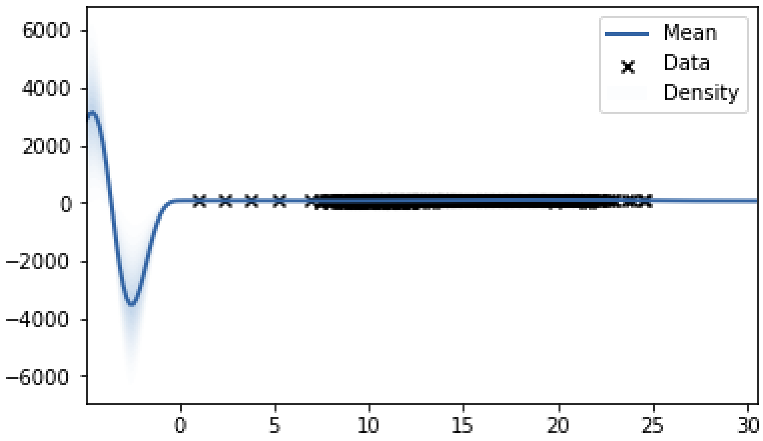

In [28]:

```
ax1.set_ylim(ax2.set_ylim(-0.1, 0.3))
ax1.set_title("GPy effect of kernel optimization")
ax1.set_ylabel("BIS Before")
ax2.set_ylabel("BIS After")
ax2.set_xlabel("HHT-IF")
fig.tight_layout()
fig.savefig("extubation_HHT-IF_BIS__p2.pdf")
```

In [29]:

```
print(gpy_model)
```

```
Name : GP regression
Objective : 3646.9595391241146
Number of Parameters : 6
Number of Optimization Parameters : 6
Updates : True
Parameters:
  GP_regression.                          |               value  |  constraints
|  priors
  mul.periodic_exponential.variance       |   49.77161628726789  |      +ve
|
  mul.periodic_exponential.lengthscale    |  170.09126146373436  |      +ve
|
  mul.periodic_exponential.period         |   19.76797273230758  |      +ve
|
  mul.Mat32.variance                      |  55.102794499750935  |      +ve
|
  mul.Mat32.lengthscale                   |  37.887654105750016  |      +ve
|
  Gaussian_noise.variance                 |   76.58130148157181  |      +ve
|
```

In [30]:

```
display(gpy_model)
```

Model: GP regression
Objective: 3646.9595391241146
Number of Parameters: 6
Number of Optimization Parameters: 6
Updates: True

GP_regression.	value	constraints	priors
mul.periodic_expone	49.77161628726789	+ve	
mul.periodic_expone	170.09126146373436	+ve	
mul.periodic_expone	19.76797273230758	+ve	
mul.Mat32.variance	55.102794499750935	+ve	
mul.Mat32.lengthsca	37.887654105750016	+ve	
Gaussian_noise.vari	76.58130148157181	+ve	

In [31]:

```
#Estimate mean and varince
x_test = np.linspace(5., 35, 300).reshape(-1, 1)
pred_mean, pred_var = gpy_model.predict(x_test.reshape(-1, 1), )
pred_std = pred_var ** .5
```

In [57]:

```
fig = plt.figure(figsize=(10,7))
plot_result(x_test, mean=pred_mean[:, 0], std=pred_std[:, 0])
plt.xlim(7, 20)
plt.ylim(20, 100)
plt.legend(['predict mean', 'training data', '1 sigma confidence'], fontsize='14')
plt.title("")
plt.xlabel('HHT-IF [Hz]', fontname='Arial', fontsize='18')
plt.ylabel('BIS', fontname='Arial', fontsize='18')
plt.savefig("extubation_HHT-IF_BIS.png", dpi=150)
fig.savefig("extubation_HHT-IF_BIS.pdf")
```

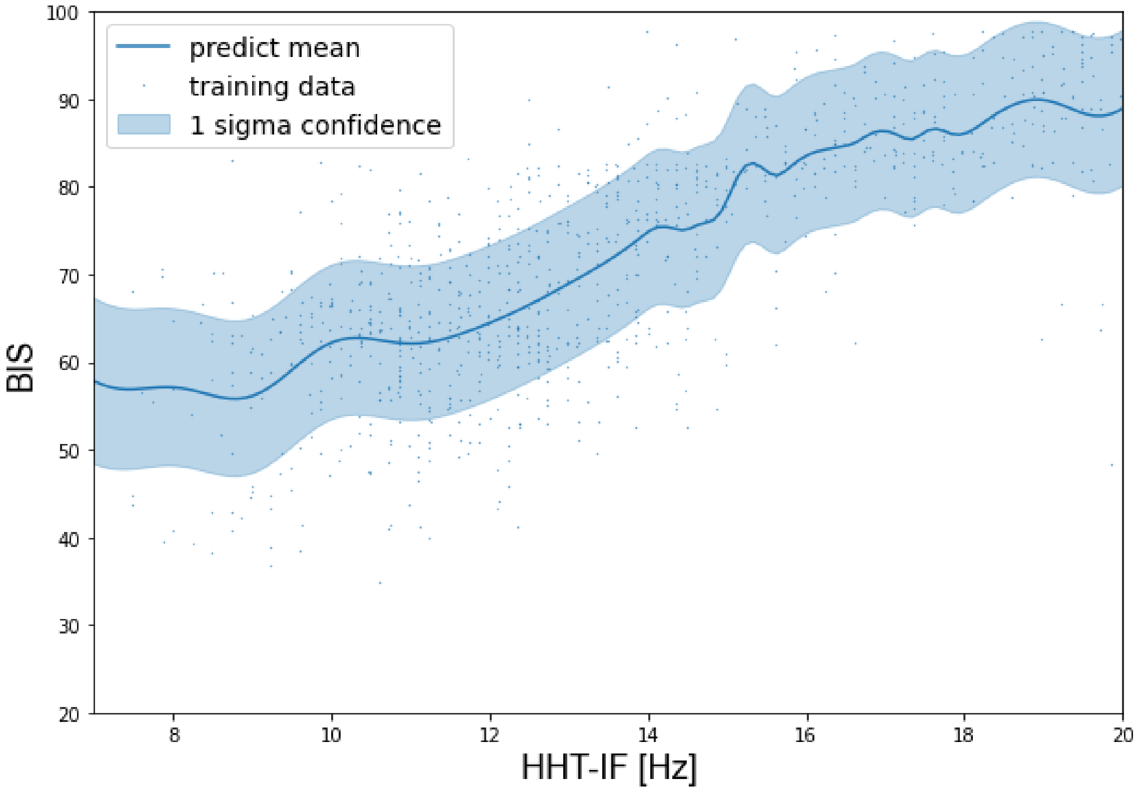

In [58]:

```
def RMSE(model, grid, ygrid):
    mu, V = model.predict(grid.reshape(-1, 1))
    Sqerr = np.power(ygrid.reshape(-1, 1) - mu,2)
    MSE = np.sum(Sqerr)
    rmse = np.sqrt(MSE/ygrid.size)
    var = np.var(ygrid.reshape(-1, 1))
    mae = np.sum(abs(ygrid.reshape(-1, 1) - mu))/ygrid.size
    print ('RMSE = ' +str(rmse))
    print ('MSE = ' +str(MSE))
    print ('MAE = ' +str(mae))
    print ('variance = ' +str(var))
    #print ('mu =:')
    #print (mu)
    #print ('V =:')
    #print (V)
    #print ('Sqerr = :')
    #print (Sqerr)
    R_square = 1- (rmse * rmse * ygrid.size)/(var * ygrid.size)
    print ('R_square =:')
    print (R_square)
    #np.savetxt("test13_mu_posterior.txt", mu, fmt='%s', delimiter=',')
    #np.savetxt("test13_V_posterior.txt", V, fmt='%s', delimiter=',')
    #np.savetxt("test13_Sqerr_posterior.txt", Sqerr, fmt='%s', delimiter=',')
    print ('Log likelihood is ' + str(model.log_likelihood()))
    return rmse
```

In [59]:

```
from sklearn.metrics import mean_absolute_error
from sklearn.metrics import mean_squared_error
from sklearn.metrics import mean_squared_log_error

def evaluate(model, grid, ygrid):
    mu, V = model.predict(grid.reshape(-1, 1))
    var = np.var(ygrid.reshape(-1, 1))
    print ('Variance =:          ' +str(var))
    rmse = np.sqrt(mean_squared_error(ygrid, mu))
    print ('RMSE =:              ' +str(rmse))
    mae = mean_absolute_error(ygrid, mu)
    print ('MAE =:               ' +str(mae))
    rmsle = np.sqrt(mean_squared_log_error(ygrid, mu))
    print ('RMSLE =             ' +str(rmsle))
    R_square = 1- (rmse * rmse * ygrid.size)/(var * ygrid.size)
    print ('R_square1 =:        ', R_square)
    R_square2 = 1-(np.mean(ygrid-mu)**2)/np.mean((ygrid-ygrid.mean())**2)
    print ('R_square2 =:        ', R_square2)

    Cov = np.mean((ygrid-mu)* (grid-grid.mean()))
    XSe = np.sqrt(np.mean((ygrid- ygrid.mean())**2))
    YSe = np.sqrt(np.mean((grid -  grid.mean())**2))

    R_square3 = Cov/(XSe*YSe)
    print ('R_square3 =:        ', R_square3)

    print ('Log likelihood =: ' + str(model.log_likelihood()))
```

In [60]:

```
evaluate(gpy_model, x_train, y_train)
```

```
Variance =:          196.03709404
RMSE =:              8.641683058044071
MAE =:                6.678491018924401
RMSLE =             0.13076473166368013
R_square1 =:         0.6190583907530876
R_square2 =:         0.9999991653937966
R_square3 =:         0.7369244024573275
Log likelihood =: -3646.9595391241146
```

In [61]:

```
def evaluate2(model, grid, ygrid):
    mu, V = model.predict(grid.reshape(-1, 1))
    var = np.var(ygrid.reshape(-1, 1))
    print ('Variance =:           ' +str(var))
    rmse = np.sqrt(mean_squared_error(ygrid, mu))
    print ('RMSE =:               ' +str(rmse))
    mae = mean_absolute_error(ygrid, mu)
    print ('MAE =:                 ' +str(mae))
    rmsle = np.sqrt(mean_squared_log_error(ygrid, mu))
    print ('RMSLE =              ' +str(rmsle))
    R_square = 1- (rmse * rmse * ygrid.size)/(var * ygrid.size)
    print ('R_square1 =:         ', R_square)
    R_square2 = 1-(np.mean(ygrid-mu)**2)/np.mean((ygrid-ygrid.mean())**2)
    print ('R_square2 =:         ', R_square2)

    Cov = np.mean((ygrid-mu)* (grid-grid.mean()))
    XSe = np.sqrt(np.mean((ygrid- ygrid.mean())**2))
    YSe = np.sqrt(np.mean((grid -  grid.mean())**2))

    R_square3 = Cov/(XSe*YSe)
    print ('R_square3 =:         ', R_square3)

    print ('Log likelihood =: ' + str(model.log_likelihood()))
```

In [63]:

```
#x_precase_2 = df[['HHT_IF_4']].values.reshape(-1,)  #induction only
#x_precase_3 = df[['HHT_IF_4']].values.reshape(-1,)  #induction only
#x_precase_4 = df[['HHT_IF_4']].values.reshape(-1,)  #induction only
x_precase_46 = df[['HHT_IF_mean_46']].values.reshape(-1,)  #extubation only
x_precase_47 = df[['HHT_IF_mean_47']].values.reshape(-1,)  #extubation
x_precase_49 = df[['HHT_IF_mean_49']].values.reshape(-1,)  #extubation
x_precase_52 = df[['HHT_IF_mean_52']].values.reshape(-1,)  #extubation
x_precase_53 = df[['HHT_IF_mean_53']].values.reshape(-1,)  #extubation
x_precase_54 = df[['HHT_IF_mean_54']].values.reshape(-1,)  #extubation
x_precase_35 = df[['HHT_IF_mean_35']].values.reshape(-1,)  #extubation
x_precase_40 = df[['HHT_IF_mean_40']].values.reshape(-1,)  #extubation
x_precase_58 = df[['HHT_IF_mean_58']].values.reshape(-1,)  #extubation
x_precase_59 = df[['HHT_IF_mean_59']].values.reshape(-1,)  #extubation

#x_case_2 = x_precase_2[~np.isnan(x_precase_2)]
#x_case_3 = x_precase_3[~np.isnan(x_precase_3)]
#x_case_4 = x_precase_4[~np.isnan(x_precase_4)]
x_case_46 = x_precase_46[~np.isnan(x_precase_46)]
x_case_47 = x_precase_47[~np.isnan(x_precase_47)]
x_case_49 = x_precase_49[~np.isnan(x_precase_49)]
x_case_52 = x_precase_52[~np.isnan(x_precase_52)]
x_case_53 = x_precase_53[~np.isnan(x_precase_53)]
x_case_54 = x_precase_54[~np.isnan(x_precase_54)]
x_case_35 = x_precase_35[~np.isnan(x_precase_35)]
x_case_40 = x_precase_40[~np.isnan(x_precase_40)]
x_case_58 = x_precase_58[~np.isnan(x_precase_58)]
x_case_59 = x_precase_59[~np.isnan(x_precase_59)]

#y_precase_2 = df[['BIS_2']].values.reshape(-1,)
#y_precase_3 = df[['BIS_3']].values.reshape(-1,)
#y_precase_4 = df[['BIS_4']].values.reshape(-1,)
y_precase_46 = df[['BIS_46']].values.reshape(-1,)
y_precase_47 = df[['BIS_47']].values.reshape(-1,)
y_precase_49 = df[['BIS_49']].values.reshape(-1,)
y_precase_52 = df[['BIS_52']].values.reshape(-1,)
y_precase_53 = df[['BIS_53']].values.reshape(-1,)
y_precase_54 = df[['BIS_54']].values.reshape(-1,)
y_precase_35 = df[['BIS_35']].values.reshape(-1,)
y_precase_40 = df[['BIS_40']].values.reshape(-1,)
y_precase_58 = df[['BIS_58']].values.reshape(-1,)
y_precase_59 = df[['BIS_59']].values.reshape(-1,)


#y_case_2 = y_precase_2[~np.isnan(y_precase_2)]
#y_case_3 = y_precase_3[~np.isnan(y_precase_3)]
#y_case_4 = y_precase_4[~np.isnan(y_precase_4)]
y_case_46 = y_precase_46[~np.isnan(y_precase_46)]
y_case_47 = y_precase_47[~np.isnan(y_precase_47)]
y_case_49 = y_precase_49[~np.isnan(y_precase_49)]
y_case_52 = y_precase_52[~np.isnan(y_precase_52)]
y_case_53 = y_precase_53[~np.isnan(y_precase_53)]
y_case_54 = y_precase_54[~np.isnan(y_precase_54)]
y_case_35 = y_precase_35[~np.isnan(y_precase_35)]
y_case_40 = y_precase_40[~np.isnan(y_precase_40)]
y_case_58 = y_precase_58[~np.isnan(y_precase_58)]
y_case_59 = y_precase_59[~np.isnan(y_precase_59)]

#print("data_2 size =:", x_case_2.size, y_case_2.size)
#print("data_3 size =:", x_case_3.size, y_case_3.size)
#print("data_4 size =:", x_case_4.size, y_case_4.size)
print("data_46 size =:", x_case_46.size, y_case_46.size)
```

```
print("data_47 size =:", x_case_47.size, y_case_47.size)
print("data_49 size =:", x_case_49.size, y_case_49.size)
print("data_52 size =:", x_case_52.size, y_case_52.size)
print("data_53 size =:", x_case_53.size, y_case_53.size)
print("data_54 size =:", x_case_54.size, y_case_54.size)
print("data_35 size =:", x_case_35.size, y_case_35.size)
print("data_40 size =:", x_case_40.size, y_case_40.size)
print("data_58 size =:", x_case_58.size, y_case_58.size)
print("data_59 size =:", x_case_59.size, y_case_59.size)

#print("")
#print("case_2 =:")
#evaluate2(gpy_model, x_case_2, y_case_2)
#print("")
#print("case_3 =:")
#evaluate2(gpy_model, x_case_3, y_case_3)
#print("")
#print("case_4 =:")
#evaluate2(gpy_model, x_case_4, y_case_4)
print("")
print("case_46 =:")
evaluate2(gpy_model, x_case_46, y_case_46)
print("")
print("case_47 =:")
evaluate2(gpy_model, x_case_47, y_case_47)
print("")
print("case_49 =:")
evaluate2(gpy_model, x_case_49, y_case_49)
print("")
print("case_52 =:")
evaluate2(gpy_model, x_case_52, y_case_52)
print("")
print("case_53 =:")
evaluate2(gpy_model, x_case_53, y_case_53)
print("")
print("case_54 =:")
evaluate2(gpy_model, x_case_54, y_case_54)
print("")
print("case_35 =:")
evaluate2(gpy_model, x_case_35, y_case_35)
print("")
print("case_40 =:")
evaluate2(gpy_model, x_case_40, y_case_40)
print("")
print("case_58 =:")
evaluate2(gpy_model, x_case_58, y_case_58)
print("")
print("case_59 =:")
evaluate2(gpy_model, x_case_59, y_case_59)
```

```
data_46 size =: 50 50
data_47 size =: 50 50
data_49 size =: 50 50
data_52 size =: 50 50
data_53 size =: 50 50
data_54 size =: 50 50
data_35 size =: 50 50
data_40 size =: 50 50
data_58 size =: 50 50
data_59 size =: 50 50

case_46 =:
Variance =:          168.14944399999996
RMSE =:              5.335269991216462
MAE =:                4.16265520524287
RMSLE =             0.06495166518736825
R_square1 =:         0.8307154326411278
R_square2 =:         0.9426510202862921
R_square3 =:         0.9511447888152756
Log likelihood =: -3646.9595391241146

case_47 =:
Variance =:          151.630256
RMSE =:              6.36946060591998
MAE =:                5.154891988592469
RMSLE =             0.08730275202451517
R_square1 =:         0.7324410742248795
R_square2 =:         0.9182355048907631
R_square3 =:         0.8975735870687898
Log likelihood =: -3646.9595391241146

case_49 =:
Variance =:          181.97977600000002
RMSE =:              9.675150835727022
MAE =:                8.051378302570368
RMSLE =             0.1537444576051073
R_square1 =:         0.48561018289159075
R_square2 =:         0.996808206775334
R_square3 =:         0.7081712328981263
Log likelihood =: -3646.9595391241146

case_52 =:
Variance =:          175.62886400000002
RMSE =:              6.052011726571235
MAE =:                4.15941414589364
RMSLE =             0.07991822699033539
R_square1 =:         0.7914531523784397
R_square2 =:         0.9547948273667276
R_square3 =:         0.8972782450314163
Log likelihood =: -3646.9595391241146

case_53 =:
Variance =:          98.131104
RMSE =:              6.76072152407316
MAE =:                5.58002844027322
RMSLE =             0.08995325129355686
R_square1 =:         0.5342215295359756
R_square2 =:         0.9832925744980741
R_square3 =:         0.8875128942196325
Log likelihood =: -3646.9595391241146
```

```
case_54 =:
Variance =:          96.22433599999998
RMSE =:              4.6879208874807565
MAE =:                3.6222803592348227
RMSLE =             0.06683557649490826
R_square1 =:         0.7716107674956741
R_square2 =:         0.9485219382753127
R_square3 =:         0.9236856670764344
Log likelihood =: -3646.9595391241146

case_35 =:
Variance =:          96.64880399999998
RMSE =:              4.7874893020521645
MAE =:                3.72971330718985
RMSLE =             0.06682474064111688
R_square1 =:         0.7628521733464604
R_square2 =:         0.9995065073662777
R_square3 =:         0.9210083264323221
Log likelihood =: -3646.9595391241146

case_40 =:
Variance =:          53.840175999999985
RMSE =:              5.068027565486829
MAE =:                4.257512374255402
RMSLE =             0.06388281542732836
R_square1 =:         0.5229416894080294
R_square2 =:         0.8324131397608359
R_square3 =:         0.859055347232072
Log likelihood =: -3646.9595391241146

case_58 =:
Variance =:          162.47027599999998
RMSE =:              7.556963442951934
MAE =:                5.743784532855694
RMSLE =             0.11372249011496902
R_square1 =:         0.6485037270564373
R_square2 =:         0.9896747545594912
R_square3 =:         0.8034581262791736
Log likelihood =: -3646.9595391241146

case_59 =:
Variance =:          85.08694399999999
RMSE =:              7.232521578267659
MAE =:                5.995976008932858
RMSLE =             0.097882685431662
R_square1 =:         0.38522450189176716
R_square2 =:         0.6320837138004392
R_square3 =:         0.7916938407508612
Log likelihood =: -3646.9595391241146
```

In [ ]:
